# Cardiac sarcoidosis: a comprehensive review of risk factors, pathogenesis, diagnosis, clinical manifestations, and treatment strategies

**DOI:** 10.3389/fcvm.2023.1156474

**Published:** 2023-05-19

**Authors:** Hussain Haider Shah, Syeda Alishah Zehra, Aliza Shahrukh, Radeyah Waseem, Tooba Hussain, Muhammad Sheheryar Hussain, Fareeha Batool, Muhammad Jaffer

**Affiliations:** Department of Internal Medicine, Dow University of Health Sciences, Karachi, Pakistan

**Keywords:** heart failure, cardiomyopathies, cardiac 1maging techniques, coronary artery disease, endovascular procedures, sarcoidosis

## Abstract

Cardiac Sarcoidosis (CS) is a deadly consequence of systemic sarcoidosis that inflames all three layers of the heart, especially the myocardium—clinical signs of CS range from asymptomatic disease to abrupt cardiac death. CS generally remains undiagnosed secondary to a lack of definitive diagnostic criteria, a high percentage of false negative results on endomyocardial biopsy, and ill-defining clinical manifestations of the disease. Consequently, there is a lack of evidence-based recommendations for CS, and the present diagnostic and therapeutic management depend on expert opinion. The aetiology, risk factors, clinical symptoms, diagnosis, and therapy of CS will be covered in this review. A particular emphasis will be placed on enhanced cardiovascular imaging and early identification of CS. We review the emerging evidence regarding the use of Electrocardiograms (ECGs), Magnetic Resonance Imaging (MRI), and Positron Emission Tomography (PET) imaging of the heart to identify and quantify the extent of myocardial inflammation, as well as to guide the use of immunotherapy and other treatment regimens, such as ablation therapy, device therapy, and heart transplantation, to improve patient outcomes.

## 1. Introduction

The official documented history of sarcoidosis dawned in the January of 1869 when John W., a 58-year-old coal wharf worker, visited Hutchinson at the Black Friars Hospital with the complaint of non-ulcerating, non-tender, purplish plaques that had slowly emerged over the past two years. Hutchinson speculated that these cutaneous lesions were a manifestation of the patient's gout and mentioned that “he came on account of colour on his extremities”(1). A Norwegian dermatologist, Caesar Boeck, was the first to coin the term “multiple, benign, skin-sarcoid” in 1899 while depicting one of the biopsied skin lesions due to its histologic resemblance to “epithelioid cells with large pale nuclei,” or a sarcoma (2). According to Scadding JG and Mitchell D., this disease is defined as the “formation of granuloma in all of several affected tissues of epithelioid-cell tubercles without caseation though fibrinoid necrosis may be present at the centre of a few, proceeding either to resolution or to conversion into hyaline fibrous tissue” (3).

CS (CS) is an essential and deadly manifestation of systemic sarcoidosis. The formation of non-caseating granulomas progresses to cause inflammation in all three layers of the heart, with the myocardium being the most prevalent location. This progression from focal inflammation to scar formation potentiates the development of cardiomyopathies and arrhythmias, leading to even cause sudden cardiac death (4). A study demonstrated that 25% of sarcoidosis patients have their myocardium affected following a subclinical course, often involving a modest portion of the myocardium (5). However, the provided statistical values cannot be generalised as the presentation of CS shows great diversity in different parts of the world.

An extensive epidemiological study of patients with CS that was conducted over more than 15 years revealed that the peak incidence of the disease occurred in both genders affecting ages ranging from 20 to 34, with older women displaying a 2nd peak that was lower but broader than the first (6). Also, the probability of developing sarcoidosis during a lifetime was 1.3% in women, but in men, the risk was over 1% (6).

It is well-established that Cardiac involvement in sarcoidosis is a poor prognostic feature (7). It is further accentuated by a subpar diagnostic rate of only 19.2% when biopsied due to the patchy involvement of the heart (8). CS can have multiple presentations in an otherwise healthy individual with no systemic symptoms.

## 2. Epidemiology

The prevalence and incidence of sarcoidosis depend on race, age and ethnicity. However, it can occur regardless of these factors. The bulk of this disease in Europe and the United States is 10–40/100,000, and compared to Caucasians, it has 3.8 times increased risk in African Americans (9, 10). Regarding incidence, it is reported to be 3.7/100,000 in Eastern Europe, 1/100,000 in Japan and 1.3/100,000 in Spain (9). The disease typically presents at an average age of 48 (9). In adults, sarcoidosis begins before age 50. Between the ages of 25 and 40, 70% of cases come to light, with a second peak of incidence occurring in women over 50 (11).

Sarcoidosis is a multisystemic disorder involving multiple organs such as the lungs, central nervous system, skin and eye. It is a surprise that the involvement of the heart is diagnosed clinically in only 5% of patients with sarcoidosis. Still, cardiac involvement is present in up to 25% of autopsy specimens when these patients undergo an autopsy. In patients with systemic sarcoidosis, the prevalence of cardiac involvement has varied from 3.7%–54.9%. The range is highly variable because of the different studied populations and other techniques (12).

## 3. Risk factors

Sarcoidosis is a granulomatous disorder of autoimmune origin and therefore shares a strong association with genetic factors. It also has a powerful coalition with environmental factors, including infectious agents, organic antigens that are not infectious, metals, and combustible materials (13, 14). Exploration of a few triggers evoked insight into occupational exposure to crystalline silica dust and a positive history of tuberculous infection (15, 16). Obesity (primarily women with BMI >30 kg/m^2^) is also enlisted as an intriguing risk factor for disease susceptibility; startlingly, current smokers have, however, a negative association with developing sarcoidosis, possibly due to suppression of T-lymphocyte function and phagocytic activity of macrophages elicited by smoking (17–19). High susceptivity to environmental triggers in some individuals compared to others lies in the genetic make-up of the individuals that make some prone to coincidental variations than others. The frequent reporting of monozygotic twins afflicted with sarcoidosis gives crude but explicit proof that genetic interplay is of consequential importance (20–22).

## 4. Pathogenesis

Sarcoidosis is a disease of unknown aetiology; it's a multisystemic, chronic inflammatory disorder characterised by non-caseating, epithelioid cell granulomas which involve many tissues and organs. In 90% of cases, it involves the lungs. Even though the left ventricle is most commonly affected in cardiac sarcoidosis, any part of the heart can be involved. The pathologic feature of the heart involves three successive histological stages: firstly, granulomatous inflammation occurs, which can cause alterations to the myocardium and possibly microcirculation, leading to temporary tissue dysfunction, tissue injury, and destruction, with or without fibrosis (23). Tissue oedema is a clinical observation that can signify the presence of granulomatous inflammation, which may be confirmed through histological examination. Imaging techniques such as MRI may detect tissue oedema as an early sign of granulomatous inflammation (24). The involvement of the heart in sarcoidosis has only been reported in 5% of cases (25), although there are 30% of patients with asymptomatic cardiac involvement (26).

The pathophysiology and aetiology of sarcoidosis still need to be fully understood. After exposure to environmental triggers, it develops in genetically predisposed individuals, triggering an exaggerated cellular immune response and eventually forming granulomas, which may resolve or progress into fibrosis (27, 28). Granulomatous inflammation develops in all layers of the heart [pericardium, myocardium, and endocardium] in a patchy multifocal pattern.

Three categories of potential etiologic factors have been found until now; these include genetic, infective and non-infective. The infectious causes include organisms such as Corynebacterium species, Mycobacterium tuberculosis, Mycoplasma species and Spirochetes. Viruses such as Herpes, Epstein-Barr, Retrovirus, Coxsackie B virus, and Cytomegalovirus have also been listed as potential antigens along with Propionibacterium acnes, Borrelia burgdorferi, drugs and environmental agents (aluminium, zirconium, hairspray, peanut dust, mineral oil, pollen, clay, talc, beryllium) (29). CD-positive T helper cells phagocytose these antigens after they've been presented to them by macrophages, leading to the formation of granuloma lesions (27, 30).

**Figure 1:**
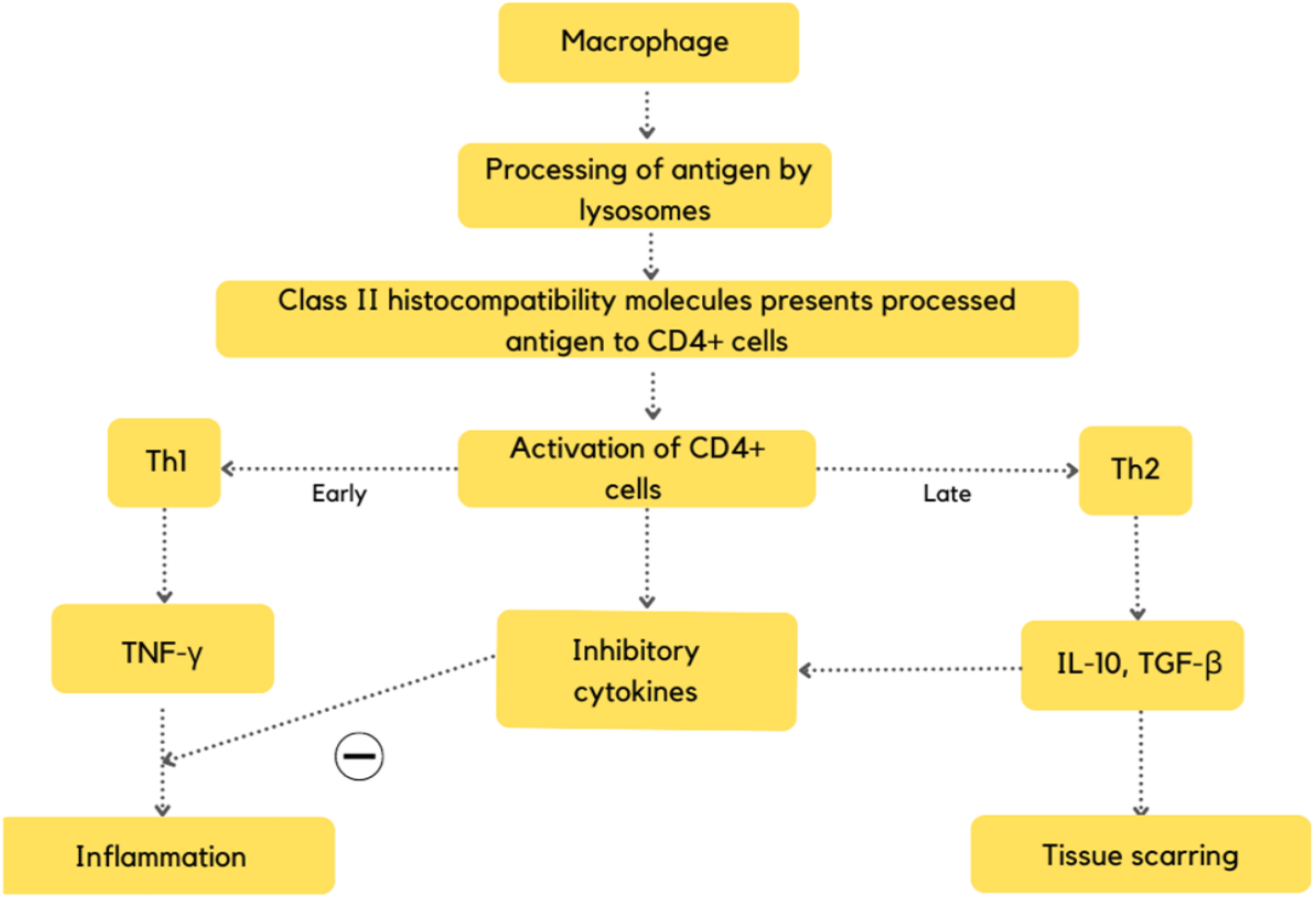
Flowchart depicting the pathogenesis of CS (27).

The cardinal immunological feature of active sarcoidosis inflammation is the dominant role of interferon-gamma resulting in Th1-related gene expression signature in the lung and blood compartments. TNF is crucial for granuloma formation. Although increased expression of the Th2 cytokine IL13 in sarcoidosis lung and blood samples compared to control has been observed, it has been hypothesised that Th2-related pathways may play a role in the later development of fibrosis; nevertheless, this remains a hypothesis and a complete switch from Th1 to Th2 has not been firmly described (Figure 1) (27).

At the onset of the disease, a high concentration of interleukin-6 was also found in the circulation. The cytokine is believed to induce the proliferation of T cells, thus maintaining inflammation (27).

Genetically susceptible individuals are more likely to develop sarcoidosis, especially individuals with specific HLA sub-types and the presence of some familial clusters. Compared to control subjects, it was observed that there is a significantly increased risk of the disease (up to five times) in first- and second-degree relatives of patients with sarcoidosis. The mode of inheritance of the risk for this disease can be polygenic, most commonly including the Class-I HLA-A1 and -B8 and class II HLADR3 genotypes. HLA-DQB1*0601 and the allele TNFA2 indicate a genetic aetiology in Japanese female patients with cardiac sarcoidosis (29).

When we look at the histopathology of cardiac sarcoidosis, we see multifocal involvement, progressing from oedema to inflammation to fibrosis and, finally, a scar. Even though the disease can involve the right ventricle and atrium, the most common site is the left ventricle (interventricular septum and papillary muscles) (31).

There's a granuloma with loose macrophages and lymphocyte dominance in early-onset cardiac sarcoidosis. As the disease worsens, the granuloma becomes more compact, and the lymphocyte decrease in number. The conditions further progress to the granulomas becoming densely fibrosed with lymphocyte dominance, suggesting that decreased granulomas are associated with worsening patient outcomes and CS due to fibrosis replacement. Still, the lack of granulomas does not exclude CS (4, 32).

## 5. Clinical presentation

The range of clinical manifestations of CS is vast and varies based on the location and size of sarcoid lesions. Typically, these symptoms may include shortness of breath, vertigo, palpitations, syncope, orthopnea, or peripheral oedema.

Pulse examination is crucial for detecting cardiac involvement in sarcoidosis patients (33).

Around 20%–50% of patients with severe sarcoidosis display the triad of bilateral hilar lymphadenopathy, erythema nodosum, and polyarthralgia (Lofgren's syndrome) (34).

Inflammatory granulomatous lesions may lead to various cardiac problems, such as myocardial infarction, conduction abnormalities, arrhythmias, recurring pericardial effusion, congestive heart failure, or sudden cardiac death (1). As cardiac involvement worsens, systolic or diastolic dysfunction may occur, causing heart failure. Heart failure symptoms include those related to fluid retention and those associated with reduced cardiac output. Symptoms such as oedema, cough, and dyspnea arise due to fluid retention. It is important to differentiate whether these symptoms are due to pulmonary or cardiac lesions in sarcoidosis patients primarily affected in the respiratory tract (35).

A review also revealed that 37% of sarcoidosis patients with cardiac involvement were asymptomatic, without any clinical signs or symptoms of the disease (36). Table 1 demonstrates that CS may manifest as arrhythmias, cardiomyopathies, and pericardial disease (37).

**Table 1:** Clinical manifestations of CS (37).

Clinical manifestations	Reported prevalence
Arrythmias	
AV block	26–62%
Bundle branch block	12–61%
Supraventricular tachycardia	0–15%
Ventricular tachycardia	2–42%
Sudden cardiac death	12–65%
Cardiomyopathy	
Congestive heart failure • Left ventricular systolic failure. • Heart failure with preserved ejection fraction or restrictive disease • Right Ventricular failure secondary to pulmonary disease	10–30%
Pericardial	
• Pericardial effusion detected by echo (common) • Pericarditis (rare)	20%

Clinical manifestations of cardiac sarcoidosis may include those associated with cardiac conduction disturbance and those associated with myocardial impairment, depending on the location and severity of granulomatous inflammation (35).

## 6. Diagnosis of cardiac sarcoidosis

CS is excluded from many differential diagnoses established due to a range of clinical manifestations, from a seemingly benign condition to sudden cardiac death (38). Around 5% of patients with sarcoidosis present clinically with symptomatic cardiac involvement. In comparison, 20%–25% of patients are asymptomatic, and among the 20%–30% of cases in pathological studies, myocardial infiltration accounted for 50% of deaths in the patients (38).

It is considered a safe clinical practice that all patients diagnosed with sarcoidosis must be screened for heart involvement, notably since the absence of extra-CS does not levy the suspicion of primary CS. Despite the condition's rarity, young patients with a history of new-onset syncope secondary to conduction system dysfunction or signs of cardiac failure should potentially be evaluated and screened for CS (39). A precise and early diagnosis is crucial to avert the liability of developing life-threatening complications by undertaking a detailed medical history, physical examination, echocardiography, and other imagining techniques as required.

Major guidelines for the diagnosis of Cardiac Sarcoidosis are as follows:

1. JCS 2016 Guideline on Diagnosis and Treatment of Cardiac Sarcoidosis (35) (Tables 2,3).
2. Guidelines from the Heart Rhythm Society (HRS 2014) (Table 4) (40).
3. Guidelines for diagnosing CS from the Japanese Ministry of Health Welfare 2006 (JMHW) (Table 5) (37).
4. World Association of Sarcoidosis and Other Granulomatous Disorders Sarcoidosis Organ (WASOG) Criteria 1999.

**Table 2:** Criteria for cardiac involvement.

Major criteria	Minor criteria
(a) High-grade atrioventricular block or fatal ventricular arrhythmia	(f) Abnormal ECG findings: Ventricular arrhythmias, bundle branch block, axis deviation, or abnormal Q waves
(b) Basal thinning of the ventricular septum or abnormal ventricular wall anatomy	(g) Perfusion defects on myocardial perfusion scintigraphy
**(c)** Left ventricular contractile dysfunction or focal ventricular wall asynergy	(h) Endomyocardial biopsy: Monocyte infiltration and moderate or severe myocardial interstitial fibrosis
(d) 67 Ga citrate scintigraphy or 18F-FDG PET reveals abnormally high tracer accumulation in the heart	
(e) Gadolinium-enhanced MRI reveals delayed contrast enhancement of the myocardium	

**Table 3:** Diagnostic guidelines for cardiac sarcoidosis.

Histological diagnosis group	Clinical diagnosis group
Positive myocardial biopsy findings	Negative myocardial biopsy findings or those not undergoing myocardial biopsy.
Cardiac sarcoidosis is diagnosed histologically when endomyocardial biopsy or surgical specimens demonstrate non-caseating epithelioid granulomas	The patient is clinically diagnosed as cardiac sarcoidosis when epithelioid granulomas are found in organs other than the heart, and clinical findings strongly suggestive of cardiac involvement are present OR when the patient shows clinical findings strongly suggestive of pulmonary or ophthalmic sarcoidosis, at least two of the five characteristic laboratory findings of sarcoidosis, and clinical findings strongly suggest cardiac involvement.

**Table 4:** The 2014 cardiac sarcoidosis diagnostic guidelines published by the heart rhythm society (HRS) (40).

Pathways to diagnosis	Criteria
Histological diagnosis	Histological diagnosis from myocardial tissue CS is diagnosed in the presence of non-caseating granuloma on histological examination of myocardial tissue with no alternative cause identified (including negative organismal stains if applicable).
	Clinical diagnosis from invasive and non-invasive studies. It is probable* that there is CS if:
Clinical diagnosis	(a) There is a histological diagnosis of extra-cardiac sarcoidosis and (b) One or more of the following is present. 1. Steroid + immunosuppressant responsive cardiomyopathy or heart block 2. Unexplained left ventricular ejection fraction<40% 3. Unexplained sustained (spontaneous or induced) ventricular tachycardia. 4. Mobitz type II second degree heart block or third-degree heart block. 5. Patchy uptake on dedicated cardiac FDG-PET (in a pattern consistent with CS) 6. Late gadolinium enhancement on CMR (in a pattern consistent with CS) 7. Positive gallium uptake (in a pattern consistent with CS) and (c) Other causes for cardiac manifestation(s) have been reasonably excluded.

CS, cardiac sarcoidosis; FDG-PET, Fluorodeoxyglucose-Positron Emission Tomography; CMR, cardiac magnetic resonance.

* In general, “probable involvement” is considered adequate to establish a clinical diagnosis of cardiac sarcoidosis.

**Table 5:** Japanese ministry of health and welfare guidelines for CS from 2006 (37).

Diagnostic category	Criteria	Comments
Histologic diagnosis	Endomyocardial biopsy demonstrates noncaseating epithelioid cell granulomata with histological or clinical diagnosis of extracardiac sarcoidosis	
Clinical diagnosis group*	• Negative endomyocardial biopsy • Presence of histologic or clinical extracardiac sarcoid	
Major clinical criteria	Advanced atrioventricular block	• Unclear what constitutes “advanced” (II? III?) or if paroxysmal block is included • First-degree block also commonly seen in CS**
	Basal thinning of the interventricular septum	• Imaging modality not specified (Echo? MR? Nuclear imaging?) • Specific thickness not specified (11 mm? 12 mm?)
	Positive cardiac gallium uptake	• Not used commonly—now routinely replaced by PE
	Depressed left ventricular ejection fraction (<50%)	• Modality not specified
Minor clinical criteria	Abnormal ECG findings	• Ventricular arrhythmias • Right bundle branch block • Axis deviation • Abnormal Q-wave • Atrial arrhythmias (sinus tachycardia or sinus exit block) commonly seen in CS excluded
	Abnormal echocardiography	• Regional wall motion abnormalities • Morphologic abnormality • Valvular abnormalities excluded • Pericardial abnormalities excluded
	Nuclear perfusion defect detected	• Reverse perfusion pattern (commonly seen) excluded
	Delayed gadolinium enhancement noted on cardiac MRI	• Localization and early enhancement not mentioned
	Endomyocardial biopsy showing interstitial fibrosis or monocyte infiltration over moderate grade	• “Moderate grade” not defined

* Requires two or more major criteria, or one major criterion and two or more minor criteria.

** CS, cardiac sarcoidosis.

**Table 6:** Comparison of CT and MRI Imaging Techniques.

	CT	MRI
Sensitivity	High	High
Ionizing radiation	Yes	No
Risk with contrast	Low	Low with good renal function
Food preparation	Not mentioned	No strict compliance required
Cardiac diagnosis	Limited	Able to differentiate and diagnose several disorders

**Table 7:** Adapted from the Heart Rhythm Society expert consensus statement, recommendations for ICD implantation in cardiac sarcoidosis patients (40).

Class	Recommendation
I	ICD implantation is recommended in patients meeting at least one of the following criteria: 1) Sustained ventricular tachycardia, including prior cardiac arrest. 2) LV EF ≤35% despite optimal medical therapy and immunosuppression (in the presence of active inflammation)
IIa	ICD implantation can be useful in patients, regardless of their LV function, meeting at least one of the following criteria: 1) Indication for permanent pacemaker implantation 2) Status after syncope or pre-syncope likely to be of arrhythmogenic etiology 3) Inducible sustained ventricular tachycardia (whether monomorphic or polymorphic) or clinically relevant ventricular fibrillation
IIb	ICD implantation may be considered in patients with LVEF in the range of 36%–49% and/or an RV ejection fraction <40%, despite optimal medical therapy for heart failure and a period of immunosuppression (if there is active inflammation).
III	ICD implantation is not recommended in patients without a history of syncope, with normal LV and right ventricular EF, absence of LGE on CMR, with negative electrophysiology study, and no indication for permanent pacing. Nonetheless, these patients should be closely monitored for their LV and right ventricular function ICD implantation is not recommended in patients meeting at least one of the following criteria: 1) Incessant ventricular tachycardias 2) Severe NYHA class IV heart failure

CMR, cardiac magnetic resonance; EF, ejection fraction; ICD, implantable cardioverter/defibrillator; LGE, late gadolinium enhancement; LV, left ventricular; NYHA, New York Heart Association.

### 6.1. JCS 2016 guideline on diagnosis and treatment of cardiac sarcoidosis

#### 6.1.1. Criteria for cardiac involvement

To diagnose cardiac sarcoidosis, cardiac findings should be evaluated using major criteria and minor criteria. The presence of the clinical conclusions satisfying either of the following strongly suggests the presence of cardiac involvement.

1. Satisfaction of two or more of the five major criteria (a)–(e).
2. Satisfaction of one of the five major criteria (a)–(e) and at least two of the three minor criteria (f)–(h).

#### 6.1.2. Diagnostic guidelines for cardiac sarcoidosis

A histological examination can diagnose cardiac sarcoidosis in patients with positive myocardial biopsy findings. However, cardiac sarcoidosis can be diagnosed clinically for patients with negative myocardial biopsy findings or without a myocardial biopsy.

### 6.2. Guidelines from the heart rhythm society (HRS 2014)

### 6.3. Guidelines for diagnosing CS from the Japanese ministry of health welfare (JMHW) 2006

## 7. Laboratory investigations

In sarcoidosis, abnormalities in blood or serum samples are not pathognomonic and do not tell us much about the disease. Clinical findings may include anaemia, decreased white blood cell count, or elevated erythrocyte sedimentation rate (ESR).

Hypercalcemia can occur due to vitamin D activation by macrophages in sarcoid granulomas, serum Immunoglobulins are elevated, and serum angiotensin-converting enzyme (ACE) levels are often raised in sarcoidosis patients (41). Therefore, ACE levels are used by some clinicians to monitor response to therapy.

Even though the rise in ACE levels in a patient may increase suspicion of sarcoidosis, the absence of an elevated ACE level is insufficient to rule out the diagnosis (41).

### 7.1. Endomyocardial biopsy

Endomyocardial biopsy serves as a gold standard for detecting CS. The current consensus by the Heart Rhythm Society (HRS), “the presence of non-caseating granuloma on histological examination of myocardial tissue with no alternative cause identified,” is the diagnostic criteria for CS (40). Despite the given diagnostic value, an endomyocardial biopsy is estimated to have less than 20% sensitivity for detecting non-caseating granulomas (8). This is believed to be a consequence of the patchy distribution of the granulomas, which may span any layer of the heart, with the myocardium most frequently involved. The region of myocardium generally occupied by granulomatous lesions is the free left ventricular wall, followed by the interventricular septum, the right ventricle, and sometimes the atrial wall.

Most sarcoid lesions in the left ventricle start in the papillary muscles and free walls of the ventricle. Most of the time, the upper half of the interventricular septum is affected rather than the lower half (4). Sarcoid granulomas tend to form in the left ventricular papillary muscles and the upper half of the interventricular septum. But small granulomas may be missed because these areas must be cut (4).

The limitations suggest that endomyocardial biopsy is of trivial value in diagnosing CS, and patients intensely suspicious of CS are treated even if the biopsy is negative (42). A study by Myung-Jin Cha et al. advised pathologists to further assess negative biopsy for a clinically probable case by presenting four presumptive indicators of CS, including microgranulomas, coalescent fibrosis, fatty changes, and increased histiocytes (42). Consequently, the authors claim that the current diagnostic criteria for CS, based on endomyocardial biopsies, need to be more conclusive when diagnosing cardiac involvement in sarcoidosis. Therefore, the authors propose that pathologic markers other than granuloma may be crucial for early clinical identification and prompting additional therapy of sarcoidosis patients.

### 7.2. Electrocardiograph

The electrocardiogram (ECG) is a hallmark in evaluating patients with sarcoidosis. Even without cardiac involvement or negative endomyocardial biopsies, 20%–50% of people with systemic sarcoidosis may have ECG changes. These changes usually show bradycardia caused by atrioventricular block (AVB), non-specific T wave abnormalities, ventricular tachyarrhythmias (VT), ventricular fibrillation (VF), and complete atrioventricular dissociation (34).

A frequent manifestation of CS is a first-degree atrioventricular block (AVB) (34). Nordenswan et al. evaluated poor prognosis in patients presenting with VT or AVB and that increased risk of sudden cardiac death during a 5-year follow-up (43). There are few concerns about the prevalence of atrial arrhythmias as they can be managed effectively by catheter ablation and has a better prognosis compared to Ventricle tachyarrhythmias which are associated with a poor prognosis and indicate an advanced level of inflammation and disease stage.

Ishiguchi et al. found that urinary 8-hydroxy-2′-deoxyguanosine strongly indicates oxidative stress. It was found to be higher in CS patients with VT and could be a good predictor for CS patients (44). A recent study of 132 patients found that the Tp–Te intervals (the time interval between the top of the T wave on an ECG and the end of the T wave) rise in people with CS, which may be the cause of arrhythmias. Early screening could help prevent patients from getting heart problems (38).

In Japan, the 12-lead-ECG at rest was looked at in 93 sarcoidosis patients to see if their hearts might be involved. They concluded that CS was linked to both bundle branch block (BBB) and T-wave amplitude in lead aVR (augmented Vector Right) (45). When these two ECG parameters are used together, it is easier to diagnose cardiac involvement of sarcoidosis with 94% sensitivity and 89% specificity compared to assessing Bundle Branch Block (BBB) alone (45).

Again, it must be understood that a normal ECG does not rule out CS.

### 7.3. Echocardiography

Echocardiography is a crucial diagnostic test for identifying cardiac sarcoidosis (CS) patients. Although primarily used to determine the cause of cardiomyopathies and ECG changes, echocardiography can also reveal subtle abnormalities in patients with sarcoidosis. In such cases, further investigation should be carried out to ascertain the possibility of CS (40).

However, echocardiography is less sensitive compared to other imaging techniques, such as positron emission tomography (PET) or cardiac magnetic resonance (CMR). Nevertheless, transthoracic echocardiogram (TTE) is the most readily available imaging modality recommended as a first-line screening tool for diagnosing CS in the updated consensus statement published by the Heart Rhythm Society, where TTE was assigned a class IIA recommendation (40).

The diagnostic accuracy of TTE ranges from 71% to 84%, as per a retrospective study of patients with biopsy-proven cardiac sarcoidosis (CS), symptoms, and Holter monitor data. However, the study found that the sensitivity of TTE alone in detecting CS is not improved, even with the presence of symptoms or Holter monitor observations (46).

Prospective studies suggest that newer indices, such as the global longitudinal strain (GLS) derived from TTE, may have an advantage in providing diagnostic and prognostic information over conventional echocardiography. For instance, a cohort of 31 patients with positive biopsy for CS and preserved left ventricular ejection fraction on TTE found that GLS was significantly reduced in patients with CS with a cutoff of −17%. This reduction in GLS showed a sensitivity and specificity of 94% for detecting CS (47).

Another study concurred that patients diagnosed recently with sarcoidosis tend to have lower GLS despite having well-maintained systolic myocardial function (48). Joyce et al. further elaborated that patients with low GLS tend to show poor prognoses, including increased mortality, device implantation, new arrhythmias, cardiac failure, or future CS development (49). Thus, patients with low GLS levels should be closely monitored for the emergence of adverse cardiac events.

### 7.4. Radionuclide scan

A powerful radioisotope tracer, 68Ga-DOTANOC, attaches itself to the special somatostatin receptors on all the inflammation mediators contributing to the formation of sarcoid granulomas aiming towards a definitive diagnosis. Prospective research conducted by Gorsemen et al. to evaluate the level of accuracy and inter-observer variability of 68Ga-DOTANOC vs. 18F-FDG PET/CT (50) suggested that A more successful option as compared to this non-specific 18F-FDG PET tracer could be somatostatin receptor (SSTR)-targeted radiotracers like 68Ga-DOTA-NaI-octreotide (DOTANOC) or 68Ga-DOTA-D-Phe-Tyr-octreotide (DOTATOC).

All the activated cells of inflammation that take part in the formation of sarcoid granulomas are packed with SSTRs on their membranes (51), particularly SSTR2A, whereas normal heart cells lack them. 68Ga-DOTANOC has been discovered to have a powerful affinity for SSTR2 and SSTR5 and does not enter the normal myocardium (52). Gallium-67 scans were frequently used in the diagnostic protocol and prognosis of CS (53); however, now it is mainly being replaced by FDG-PET scans (54).

In another case, Biventricular SPECT with technetium-99 m was used as a clinically useful tool for the noninvasive assessment of both ventricles in a patient with sarcoidosis (55). Using two imaging techniques like, Tc 99 m MIBI and Ga-68DOTANOC PET–CT is also fruitful in confirming suspected CS (56). Another significant support in this labyrinth of CS is provided by Thallium Myocardial Perfusion Imaging, which was very helpful in monitoring response to treatment, as in this case, it could not be done by MR because of the ICD (57).

### 7.5. Cardiac magnetic resonance imaging (CMR)

Cardiac magnetic resonance (CMR) imaging involving the specific late gadolinium enhancement was introduced in 1989 (58) to localise dead or damaged myocardial tissue. The large molecular size of Gadolinium permits it to accumulate in the extracellular spaces without entering the cardiomyocytes under normal circumstances. However, under specific pathologies which may lead to inflammation and scar formation, as in CS, the amount of Gad collecting in the ECS may increase, or a breach in the cell membrane may be disturbed. All this will eventually lead towards a detectable rise in Gadolinium's distribution volume and subsequently cause enhancement.

A study evaluating Cardiac MRI indicated GadCMR is potentially superior to the Criteria for the diagnosis of CS established by the Japanese Ministry of Health and Welfare (JMHW) (59). The myocardial lesions identified on delayed contrast-enhanced MRI were confined in the basal and subepicardial myocardium. Also, Left ventricular dysfunction and plasma BNP levels may correlate with the extent of myocardial damage in CS (60). Besides Late Gadolinium enhancement (LGE), there is a rising interest in combining T2 mapping to CMR to encircle all possible areas of reversible myocardial damage (i.e., cardinal signs of inflammation), to amplify the localisation of active CS and note down beforehand the increased risk of ventricular arrhythmias (61). It has also been found that hyperenhancement is often linked to a decrease in regional wall motion and thallium-201 perfusion defects (62). In an extensive study of 37,788 patients, only 45 suffered from adverse reactions related to Gadolinium. However, on a larger scale, the study concluded that gadolinium contrast CMR is known to have a shallow risk in patients who have moderately good renal function (GFR > 30 ml/min) (63). It is known that the element gadolinium gets deposited in the brain in patients undergoing this procedure in very minute amounts. A recent study on a rat model indicated no neurotoxicity after exposure to Gadolinium-based linear and macrocyclic contrast mediums (64).

Smedema et al. worked over to calculate the role of CMRI in 58 patients suspected to have CS. Their evaluation shows Delayed Enhancement (DE) ‘s sensitivity and specificity were 100% and 78%, respectively (65). In addition to the reliability of DE in assessing the disease, it is also a powerful tool for evaluating the response to steroid therapy (66). Absolute contraindications of CMRI include Allergy to the contrast medium, non-MRI compatible implants/foreign bodies, e.g., cardiac pacemakers, large pieces of shrapnel, Cochlear implants/ear implants and Cerebral artery aneurysm clips etc.

### 7.6. Positron emission tomography scan

Fluorodeoxyglucose positron emission tomography, or f-FDG PET, is a relatively safe non-penetrating imaging procedure that is peculiarly responsive to rapidly dividing cells (malignant or non-malignant). The isotope, as mentioned earlier, enters the metabolically active cells through the membrane glucose transporters (GLUT) and is further phosphorylated to form a compound called FDG-6 phosphate. The idea here is that the cellular enzymes do not metabolise this newly formed compound, which results in their steady accumulation inside. This simple phenomenon is termed metabolic trapping. Their high glycolytic activity can readily pick up cells exhibiting any inflammatory reaction. The flag-bearers of inflammation, like neutrophils and monocytes, recruit GLUT1 and GLUT3 receptors to the cell membrane. Also, there is a reasonably predictable rise in the levels of hexokinase compared to normal cells (67, 68).

Localised retention in the cardiomyocytes, as visualised on 18F-FDG PET images, is a classic feature of patients with sarcoidosis and key to a definitive diagnosis (69). The sensitivity of 18F-FDG, when employed for detecting CS, is high compared to its specificity, which ranges from 39%–97%. One plausible reason for this can be attributed to the fact that there is some degree of non-specific uptake by the normal heart muscle (70). Tadamura et al. recorded a case in which there was a dramatic fall in the regional uptake of 18F-FDG after steroid therapy. On top of that, there was a successful decrease in the serum levels of Angiotensin Converting Enzyme (ACE), which may correspond to a decreased disease burden (71). Similarly, Takeda et al. brought forward a case of CS complicated with third-degree AV block.

The patient had remarkably high levels of 18FFDG uptake in the basal interventricular septum. After undergoing full-blown steroid therapy, the myocardial uptake levels dwindled, and our previous 3rd-degree AV block significantly improved and turned into a 1st-degree block (72). There are no approved guidelines or standard protocols for suppressing the non-specific 18F-FDG uptake by the cardiac muscle. Nevertheless, a recent proposal has managed to gather the significant factors, which include prolonged fasting (73), dietary changes (74, 75), and unfractionated heparin load before the procedure (76).

There is one crucial drawback worth mentioning here, steroids are known to increase blood sugar levels by promoting gluconeogenesis and insulin resistance. This problematic side effect of steroid therapy can potentially disturb the 18F-FDG uptake in our target organs (77). Table 6 shows comparison of CT and MRI Imaging Techniques.

## 8. Treatment of cardiac sarcoidosis

Untreated CS has a paramount death rate; despite this, symptomatic sarcoidosis diagnosis is not well established. CS treatment should begin swiftly even if no signs of arrhythmias or left ventricular dysfunction are present for a better outlook. Patients' prognoses worsen dramatically on a delay of treatment as they develop symptoms of systolic or diastolic dysfunction leading to heart failure (78).

The two main pillars of CS treatment are:

1. The Medical management includes immunosuppressant therapy, anti-arrhythmic drugs, and guideline-based medical therapy for treating cardiac failure, and
2. Invasive treatment encompasses implantable cardioverter defibrillator implantation, Catheter Ablation, Device therapy, and Heart transplantation (79).

### 8.1. Medical management

#### 8.1.1. Corticosteroids

Although the specific treatment for CS is unknown owing to a lack of large cohorts and RCTs, Corticosteroids are still considered the backbone of therapy due to their effects in reducing the inflammation and fibrosis seen in sarcoidosis (40, 80).

The action of steroids is by suppressing granuloma formation via inhibition of production of the interplaying cytokines, functional restoration of CD4+ T cells, and balancing effector CD4+ T cells subtypes (81, 82).

Although the ideal corticosteroid dosage for treating CS has not yet been determined, the Japanese Circulation Society recommends starting Prednisone at 30 mg daily or 60 mg every other day for four weeks. The amount must be tapered to 5 mg monthly to reach a maintenance dose of 5 mg daily or 10–20 mg on alternate days for 6 months (35).

Corticosteroids provide early symptomatic relief and normalise Acetylcholinesterase (ACE) and lysozyme levels (83). A study revealed that steroid administration reduces the frequency of premature ventricular beats and non-sustained ventricular tachycardia in patients with preserved cardiac function (84). A clinical trial concluded that corticosteroids are valuable in treating CS complicated by AV block; however, monitoring is required while lowering the maintenance dose (85). Patients with LVEF <54% showed a substantial reduction in Left Ventricular volumes and demonstrated improvement in Left Ventricular Ejection Fraction following corticosteroid treatment, whereas patients with LVEF <30% exhibited no change in LV volume or function (34).

This data shows that corticosteroid medication may be protective or therapeutic in the early or middle stages—however, the effects vane in the advanced stages of illness (34). Consequential side effects are also linked to corticosteroid usage, including increased risk for venous thromboembolism (86).

#### 8.1.2. Other immunosuppressive regimens

Immunosuppressants are given to people who don't respond well to corticosteroids or who can't handle the side effects of corticosteroids. Azathioprine, Methotrexate, Leflunomide, mycophenolate mofetil, cyclosporine or cyclophosphamide may be used in individuals who do not respond adequately to corticosteroids (80). These agents lower the risk of fatal complications in CS. Methotrexate is considered an adjuvant for sarcoidosis in cases where steroid therapy is inefficacious (27). Methotrexate modulates the cells' function in inflammatory processes and inhibits folate-dependent new synthesis of nitrogenous bases, which are inevitable elements for inflammatory cellular replication. There are chances that adenosine levels will be enhanced with methotrexate, elevated extracellular adenosine levels, which may have anti-inflammatory effects (87).

A study compared the patients of CS treated with either combination therapy (Methotrexate and low dose corticosteroid (5–15 mg) or single-agent steroid therapy showed that Left Ventricular Ejection Fraction and Brain Natriuretic Peptide (BNP) were more stable when patients were treated with combination group (88). WASOG recommends starting treatment with 5–15 mg per week and increasing it by 5 mg every 2–4 weeks up to a maximum of 20 mg (89). Regular checks are necessary because of the risk of hepatotoxicity and hematologic toxicity. Monitoring renal function is also required since the medication is eliminated via the kidneys. In the case of kidney problems, the dose may need to be changed, or a different drug may need to be used. Every one to three months, a patient should have a complete blood count (CBC) and a liver and kidney function test.

To reduce the toxic effects of methotrexate, doctors may recommend taking folic acid supplements (90).

#### 8.1.3. Anti-arrhythmic drugs (AAD)

Conduction abnormalities, ventricular tachycardia, and heart failure are the three components that make up the triad of CS. Ventricular tachycardia secondary to CS can be challenging to control and could not be prevented by steroids. When active inflammation is evident, corticosteroids and anti-arrhythmic drugs should be initiated for VTs.

Limited data are available on anti-arrhythmic medications in CS. Beta-blockers and Class III anti-arrhythmic such as Amiodarone, dofetilide, and sotalol are classic choices for managing atrial and ventricular arrhythmias, although VTs are frequently resistant. Amiodarone and sotalol help treat ventricular arrhythmias resistant to immunosuppressive treatment (40). The potential adverse effects of Amiodarone include Pulmonary fibrosis and pneumonitis, so its long-term treatment is not indicated in young patients (40, 91).

It is reported that Class I anti-arrhythmic agents should be avoided in patients with myocardial scarring and structural heart disease (92).

#### 8.1.4. Biological therapy

Biological Therapies mitigate the physiological behaviour of our immune system against a pathogenic antigen. These antibodies are driven from an external animal source sensitised to a peculiar antigen or are prepared artificially in the laboratory, hence being monoclonal.

Patients refractory to corticosteroid and methotrexate therapy are incidentally treated with Biological Therapy encompassing TNF-α inhibitors (infliximab, Adalimumab, golimumab, and etanercept). Their efficacy is debatable, with significant discontinuations and adversities reported, with rates as high as 23% and 56% (93).

TNF is instrumental in developing and maintaining granulomas; hence TNF-α inhibitors are anticipated to play a significant role in their regression. Infliximab and Adalimumab are the top choices administered in patients screened negative for Hepatitis B, C, and HIV. Latent TB must also be ruled out before initiating therapy. Table 7 Adapted from the Heart Rhythm Society expert consensus statement, recommendations for ICD implantation in cardiac sarcoidosis patients.

Infliximab is administered at weeks 0, 2, 6, and every 4 to 8 weeks at 3 to 5 mg/kg body weight. Adalimumab is directed at 80 to 160 mg at week 0, followed by 40 mg week 1, and 40 mg weekly hence after. Regular monitoring for heart failure exacerbation, hypersensitivity reactions, and aggravation of infections and malignancy is crucial. The drugs must be avoided in patients recumbent to demyelinating diseases to prevent worsening conditions.

A complete work-up of the patients, including all baseline tests and chest radiographic imaging, and completion of vaccination regimens must be ensured before treatment initiation (94).

### 8.2. Invasive management

#### 8.2.1. Device therapy

Implantable cardioverter-defibrillators (ICDs) are employed for primary and secondary prevention of sudden cardiac death in patients with CS and who have developed ventricular tachycardias and ventricular fibrillation (95). As CS patients are at risk of increased incidence of malignant arrhythmias and sudden cardiac death, the Heart rhythm society expert consensus has proposed guidelines for ICD implantation. ICDs are recommended for people with optimal medical treatment but still have sustained ventricular arrhythmias or a left ventricular ejection fraction of less than 35%. ICDs can help people who have CS, regardless of how well their ventricles work, and one or more of the following: (1) Syncope or near-syncope that can't be explained and is thought to be caused by an irregular heartbeat. (2) Inducible ventricular arrhythmias (430 s of monomorphic VT or polymorphic VT) or clinically significant VF. Table 4 gives more information about ICD implantation for people with CS (40).

#### 8.2.2. Ablation therapy

One of the direct complications of CS is ventricular arrhythmias, a strong mortality predictor (96). In Around 50% of the CS patients who develop ventricular tachycardias, medical treatment is ineffective. Patients with CS who have not responded to anti-arrhythmic and immunosuppressive medication frequently benefit from radiofrequency catheter ablation of VT to completely eradicate VT or significantly reduce the VT burden. Higher left ventricular ejection fractions provided better ablation results, probably due to a more confined arrhythmogenic substrate than individuals with moderate to severe left ventricular dysfunction (97). In Table 8, Suggestions for categorising risk, averting sudden cardiac death, and managing ventricular arrhythmias in patients with cardiac sarcoidosis are Stated.

**Table 8:** Recommendations for risk stratification, sudden cardiac death prevention, and treatment of ventricular arrhythmias in cardiac sarcoidosis (98).

Recommendations	Class ^a^	Level ^b^
Risk stratification and primary prevention of SCD		
ICD implantation is recommended in patients with cardiac sarcoidosis who have a LVEF ≤35%.	I	B
In patients with cardiac sarcoidosis who have an indication for permanent cardiac pacing related to high-degree AV block, ICD implantation should be considered, regardless of LVEF.	IIa	C
In patients with cardiac sarcoidosis who have a LVEF >35% but significant LGE at CMR after resolution of acute inflammation, ICD implantation should be considered.	IIa	B
In patients with cardiac sarcoidosis who have a LVEF 35%–50% and minor LGE at CMR, after resolution of acute inflammation, PES for risk stratification should be considered.	IIa	C
In patients with cardiac sarcoidosis, LVEF 35%–50% and inducible SMVT at PES, ICD implantation should be considered.	IIa	C
Secondary prevention of SCD and treatment of VAs		
ICD implantation is recommended in patients with cardiac sarcoidosis who (1) have documented sustained VT, or (2) aborted CA.	I	B
In patients with cardiac sarcoidosis and recurrent, symptomatic VA, AAD treatment should be considered.	IIa	C
Catheter ablation, in specialized centers, may be considered in cardiac sarcoidosis ICD-recipients with recurrent, symptomatic SMVT or ICD shocks for SMVT, in whom AADs are ineffective, contraindicated, or not tolerated.	IIb	C

AAD, anti-arrhythmic drug; AV, atrio-ventricular; CA, cardiac arrest; CMR, cardiac magnetic resonance; ICD, implantable cardioverter defibrillator; LGE, late gadolinium enhancement; LVEF, left ventricular ejection fraction; PES, programmed electrical stimulation; SCD, sudden cardiac death; SMVT, sustained monomorphic VT; AV, ventricular arrhythmia; VT, ventricular tachycardia.

^a^ Class of recommendation.

^b^ Level of evidence.

#### 8.2.3. Heart transplantation

Heart transplantation is an excellent way to treat end-stage heart disease caused by CS that doesn't get better with medication. Resistant ventricular tachyarrhythmias and heart failure, especially in younger people, are the main reasons people need a heart transplant (34). Short- and long-term outcomes are identical to other cardiomyopathies (99). It is also thought that Corticosteroid treatment before severe systolic failure may prevent heart transplantation (34). Recurrence of sarcoidosis in the transplanted heart is a concern because the disease has recurred 24 to 19 months after transplantation (100).

## 9. Conclusion

Heart involvement is a potentially life-threatening complication of sarcoidosis, making it crucial to detect and diagnose the disease as early as possible. Recent research indicates that cardiac sarcoidosis (CS) may manifest as the earliest sign of sarcoidosis, highlighting the importance of early detection. Fortunately, advancements in diagnostic techniques have greatly improved our ability to detect and classify the severity of the disease. Sophisticated imaging techniques such as cardiac MRI and 18F-FDG PET can now see CS in patients at earlier stages of the disease. However, despite these advancements, confirming a diagnosis and initiating treatment for CS remains a slow process, even in patients suspected of having the condition.

While corticosteroids are effective in suppressing inflammation and preventing organ failure, the optimal duration of therapy and the relevance of second-line treatments are still unknown. As a result, diagnosing and managing CS continues to be challenging for physicians. Multi-centre research efforts are underway to better understand the disease and its treatment, but much work remains to be done. Early detection and prompt treatment remain crucial to improving outcomes for patients with cardiac sarcoidosis.

## References

[1] ‘Hutchinson J. Illustrations of clinical surgery. London: J. & A. Churchill (1875). Vol. 2. Available at: https://archive.org/details/b21515736_0002 (Cited September 29, 2022).

[2] Danbolt N. The historical aspects of sarcoidosis. Postgrad Med J. (1958) 34(391):245–67. 10.1136/pgmj.34.391.245 13553999 PMC2501582

[3] Scadding JG Mitchell DN. The immunology of sarcoidosis. In: Sarcoidosis. 2nd ed. Boston, MA: Springer US (1985). p. 36–42.

[4] Roberts WC McAllister HA Ferrans VJ. Sarcoidosis of the heart. Am J Med. (1977) 63(1):86–108. 10.1016/0002-9343(77)90121-8 327806

[5] Silverman KJ Hutchins GM Bulkley BH. Cardiac sarcoid: a clinicopathologic study of 84 unselected patients with systemic sarcoidosis. Circulation. (1978) 58(6):1204–11. 10.1161/01.CIR.58.6.1204 709777

[6] Hillerdal G Nöu E Osterman K Schmekel B. Sarcoidosis: epidemiology and prognosis. A 15-year European study. Am Rev Respir Dis. (1984) 130(1):29–32. 10.1164/arrd.1984.130.1.29 6742607

[7] Bernstein M. Boeck’s sarcoid. Arch Intern Med. (1929) 44(5):721. 10.1001/archinte.1929.00140050098009

[8] Uemura A Sichiro M Hiramitsu S Kato Y Ito T Hishida H. Histologic diagnostic rate of CS: evaluation of endomyocardial biopsies. Am Heart J. (1999) 138(2):299–302. 10.1016/S0002-8703(99)70115-8 10426842

[9] Llanos O Hamzeh N. Sarcoidosis. Med Clin N Am. (2019) 103(3):527–34. 10.1016/j.mcna.2018.12.011 30955519

[10] Rybicki BA Major M Popovich J Maliank MJ lannuzzi MC. Racial differences in sarcoidosis incidence: a 5-year study in a health maintenance organization. Am J Epidemiol. (1997) 145(3):234–41. 10.1093/oxfordjournals.aje.a009096 9012596

[11] Costabel U Hunninghake GW, on behalf of the Sarcoidosis Statement Committee. ATS/ERS/WASOG statement on sarcoidosis. Eur Respir J. (1999) 14(4):735. 10.1034/j.1399-3003.1999.14d02.x 10573213

[12] Hulten E Aslam S Osborne M Abbasi S Bittencourt MS Blankstein R. CS-state of the art review. Cardiovasc Diagn Ther. (2016) 6(1):50–63. 10.3978/j.issn.2223-3652.2015.12.13 26885492 PMC4731586

[13] Starshinova AA Malkova AM Basantsova NY Zinchenko YS Kudryavtsev Iv Ershov GA Sarcoidosis as an autoimmune disease. Front Immunol. (2020) 10. 10.3389/fimmu.2019.02933 31969879 PMC6960207

[14] Judson MA. Environmental risk factors for sarcoidosis. Front Immunol. (2020) 11. 10.3389/fimmu.2020.01340 PMC7333358 32676081

[15] Hoy RF Chambers DC. Silica-related diseases in the modern world. Allergy. (2020) 75(11):2805–17. 10.1111/all.14202 31989662

[16] Wang S Chung C Huang T Tsai W Peng C Huang K Bidirectional association between tuberculosis and sarcoidosis. Respirology. (2019) 24(5):467–74. 10.1111/resp.13482 30722101

[17] Dumas O Boggs KM Cozier YC Stampfer MJ Camargo CA. Prospective study of body mass index and risk of sarcoidosis in US women. Eur Respir J. (2017) 50(4):1701397. 10.1183/13993003.01397-2017 29051275 PMC5714277

[18] Ungprasert P Crowson CS Matteson EL. Smoking, obesity and risk of sarcoidosis: a population-based nested case-control study. Respir Med. (2016) 120:87–90. 10.1016/j.rmed.2016.10.003 27817820 PMC5185320

[19] W M. Smoking impairs alveolar macrophage activation after inert dust exposure. Toxicol Lett. (1996) 88(1–3):131–7. 10.1016/0378-4274(96)03728-9 8920727

[20] Yoshikawa T Yamamoto M Inaba S Nagai T Terai T. Sarcoidosis in identical twins. Nihon Kyobu Shikkan Gakkai Zasshi. (1994) 32(6):610–5. . 8089953

[21] Kneitz C Wilhelm M Kraus MR Tony HP Tschammler A Jany B. Sarkoidose bei eineiigen zwillingen. Dtsch Med Wochenschr. (2008) 120(24):867–73. 10.1055/s-2008-1055419 7796723

[22] Rogers FJ. Sarcoidosis in identical twins. J Am Med Assoc. (1954) 155(11):974. 10.1001/jama.1954.73690290007006d 13162839

[23] Ohira H Tsujino I Ishimaru S Oyama N Takei T Tsukamoto E Myocardial imaging with 18F-fluoro-2-deoxyglucose positron emission tomography and magnetic resonance imaging in sarcoidosis. Eur J Nucl Med Mol Imaging. (2008) 35(5):933–41. 10.1007/s00259-007-0650-8 18084757

[24] Shah KK Pritt BS Alexander MP. Histopathologic review of granulomatous inflammation. J Clin Tuberc Other Mycobact Dis. (2017) 7:1–12. 10.1016/j.jctube.2017.02.001 31723695 PMC6850266

[25] Perry A Vuitch F. Causes of death in patients with sarcoidosis. A morphologic study of 38 autopsies with clinicopathologic correlations. Arch Pathol Lab Med. (1995) 119(2):167–72. . 7848065

[26] Iannuzzi MC Rybicki BA Teirstein AS. Sarcoidosis. N Engl J Med. (2007) 357(21):2153–65. 10.1056/NEJMra071714 18032765

[27] Doughan AR. Cardiac sarcoidosis. Heart. (2006) 92(2):282–8. 10.1136/hrt.2005.080481 16415205 PMC1860791

[28] Culver DA Newman LS Kavuru MS. Gene-environment interactions in sarcoidosis: challenge and opportunity. Clin Dermatol. (2007) 25(3):267–75. 10.1016/j.clindermatol.2007.03.005 17560304 PMC1920704

[29] Ipek E Demirelli S Ermis E Inci S. Sarcoidosis and the heart: a review of the literature. Intractable Rare Dis Res. (2015) 4(4):170–80. 10.5582/irdr.2015.01023 26668777 PMC4660858

[30] Statement on sarcoidosis. Am J Respir Crit Care Med. (1999) 160(2):736–55. 10.1164/ajrccm.160.2.ats4-99 10430755

[31] Sharma A Okada DR Yacoub H Chrispin J Bokhari S. Diagnosis of CS: an era of paradigm shift. Ann Nucl Med. (2020) 34(2):87–93. 10.1007/s12149-019-01431-z 31848928

[32] Lagana SM Parwani Av Nichols LC. CS: a pathology-focused review. Arch Pathol Lab Med. (2010) 134(7):1039–46. 10.5858/2009-0274-RA.1 20586635

[33] Mehta D Lubitz SA Frankel Z Wisnivesky JP Einstein AJ Goldman M Cardiac involvement in patients with sarcoidosis. Chest. (2008) 133(6):1426–35. 10.1378/chest.07-2784 18339784

[34] Sekhri V Sanal S DeLorenzo LJ Aronow WS Maguire GP. CS: a comprehensive review. Arch Med Sci. (2011) 4:546–54. 10.5114/aoms.2011.24118 PMC3258766 22291785

[35] Terasaki F Azuma A Anzai T Ishizaka N Ishida Y Isobe M JCS 2016 Guideline on diagnosis and treatment of cardiac sarcoidosis- digest version. Circ J. (2019) 83(11):2329–88. 10.1253/circj.CJ-19-0508 31597819

[36] Mankad P Mitchell B Birnie D Kron J. CS. Curr Cardiol Rep. (2019) 21(12):152. 10.1007/s11886-019-1238-1 31768666 PMC7092931

[37] Houston BA Mukherjee M. CS: clinical manifestations, imaging characteristics, and therapeutic approach. Clin Med Insights Cardiol. (2014) 8s1:CMC.S15713. 10.4137/CMC.S15713 PMC4240214 25452702

[38] Emet S Onur S Sokucu S Aydin S Dalar L Cetinkaya E A new electrocardiographic parameter associated with sudden cardiac death in pulmonary sarcoidosis. Arch Med Sci. (2020) 16(3):559–68. 10.5114/aoms.2019.88393 32399103 PMC7212222

[39] Mantini N Williams B Jr Stewart J Rubinsztain L Kacharava A. Cardiac sarcoid: a clinician’s review on how to approach the patient with cardiac sarcoid. Clin Cardiol. (2012) 35(7):410–5. 10.1002/clc.21982 22499155 PMC6652737

[40] Birnie DH Sauer WH Bogun F Cooper JM Culver DA Duvernoy CS HRS expert consensus statement on the diagnosis and management of arrhythmias associated with CS. Heart Rhythm. (2014) 11(7):1304–23. 10.1016/j.hrthm.2014.03.043 24819193

[41] Kahkouee S Samadi K Alai A Abedini A Rezaiian L. Serum ACE level in sarcoidosis patients with typical and atypical HRCT manifestation. Pol J Radiol. (2016) 81:458–61. 10.12659/PJR.897708 27733890 PMC5036380

[42] Cha MJ Seo JW Oh S Park EA Lee SH Kim MY Indirect pathological indicators for CS on endomyocardial biopsy. J Pathol Transl Med. (2020) 54(5):396–410. 10.4132/jptm.2020.06.10 32717775 PMC7483025

[43] Nordenswan HK Lehtonen J Ekström K Kandolin R Simonen P Mäyränpää M Outcome of CS presenting with high-grade atrioventricular block. Circ Arrhythm Electrophysiol. (2018) 11(8. 10.1161/CIRCEP.117.006145 30354309

[44] Ishiguchi H Kobayashi S Myoren T Kohno M Nanno T Murakami W Urinary 8-hydroxy-2′-deoxyguanosine as a myocardial oxidative stress marker is associated with ventricular tachycardia in patients with active CS. Circ Cardiovasc Imaging. (2017) 10(12. 10.1161/CIRCIMAGING.117.006764 29208596

[45] Tanaka Y Konno T Yoshida S Tsuda T Sakata K Furusho H T wave amplitude in lead aVR as a novel diagnostic marker for CS. Heart Vessels. (2017) 32(3):352–8. 10.1007/s00380-016-0881-3 27465594

[46] Kouranos V Tzelepis GE Rapti A Mavrogeni S Aggeli K Douskou M Complementary role of CMR to conventional screening in the diagnosis and prognosis of CS. JACC Cardiovasc Imaging. (2017) 10(12):1437–47. 10.1016/j.jcmg.2016.11.019 28330653

[47] Murtagh G Laffin LJ Patel Kv Patel Av Bonham CA Yu Z Improved detection of myocardial damage in sarcoidosis using longitudinal strain in patients with preserved left ventricular ejection fraction. Echocardiography. (2016) 33(9):1344–52. 10.1111/echo.13281 27677642 PMC5523448

[48] Aggeli C Felekos I Tousoulis D Gialafos E Rapti A Stefanadis C. Myocardial mechanics for the early detection of CS. Int J Cardiol. (2013) 168(5):4820–1. 10.1016/j.ijcard.2013.07.010 23870643

[49] Joyce E Ninaber MK Katsanos S Debonnaire P Kamperidis V Bax JJ Subclinical left ventricular dysfunction by echocardiographic speckle-tracking strain analysis relates to outcome in sarcoidosis. Eur J Heart Fail. (2015) 17(1):51–62. 10.1002/ejhf.205 25431267

[50] Gormsen LC Haraldsen A Kramer S Dias AH Kim WY Borghammer P. A dual tracer 68Ga-DOTANOC PET/CT and 18F-FDG PET/CT pilot study for detection of CS. EJNMMI Res. (2016) 6(1):52. 10.1186/s13550-016-0207-6 27316444 PMC4912521

[51] Retraction: “An ∼400 kDa membrane-associated complex that contains one molecule of the resistance protein Cf-4’ by Susana Rivas, Tatiana Mucyn, Harrold A. van den Burg, Jacques Vervoort and Jonathan D. G. Jones. Plant J. (2017) 29(6):783–96. 10.1111/tpj.13577 12148536

[52] Prasad V Baum RP. Biodistribution of the Ga-68 labeled somatostatin analogue DOTA-NOC in patients with neuroendocrine tumors: characterisation of uptake in normal organs and tumor lesions. Q J Nucl Med Mol Imaging. (2010) 54(1):61–7. 20168287

[53] Futamatsu H Suzuki J Adachi S Okada H Otomo K Ohara T Utility of gallium-67 scintigraphy for evaluation of CS with ventricular tachycardia. Int J Cardiovasc Imaging. (2006) 22(3–4):443–8. 10.1007/s10554-005-9043-x 16763884

[54] Kouranos V Wells AU Sharma R Underwood SR Wechalekar K. Advances in radionuclide imaging of CS. Br Med Bull. (2015) 115(1):151–63. 10.1093/bmb/ldv033 26311504

[55] Eguchi M Tsuchihashi K Hotta D Hashimoto A Sasao H Yuda S Technetium-99 m sestamibi/tetrofosmin myocardial perfusion scanning in cardiac and NonCS. Cardiology. (2000) 94(3):193–9. 10.1159/000047316 11279326

[56] Passah A Kaushik P Patel C Parakh N. Gallium-68 DOTANOC scan in a patient with suspected CS. J Nucl Cardiol. (2018) 25(6):2177–8. 10.1007/s12350-017-1178-3 29327255

[57] Surasi DS Manapragada PP Lloyd SG Bhambhvani P. Role of multimodality imaging including thallium-201 myocardial perfusion imaging in the diagnosis and monitoring of treatment response in CS. J Nucl Cardiol. (2014) 21(4):849–52. 10.1007/s12350-014-9861-0 24493414

[58] Saeed M Wagner S Wendland MF Derugin N Finkbeiner WE Higgins CB. Occlusive and reperfused myocardial infarcts: differentiation with mn-DPDP–enhanced MR imaging. Radiology. (1989) 172(1):59–64. 10.1148/radiology.172.1.2500678 2500678

[59] Manins V Habersberger J Pfluger H Taylor AJ. Cardiac magnetic resonance imaging in the evaluation of CS: an Australian single-centre experience. Intern Med J. (2009) 39(2):77–82. 10.1111/j.1445-5994.2008.01674.x 18771431

[60] Ichinose A Otani H Oikawa M Takase K Saito H Shimokawa H MRI of CS: basal and subepicardial localization of myocardial lesions and their effect on left ventricular function. Am J Roentgenol. (2008) 191(3):862–9. 10.2214/AJR.07.3089 18716120

[61] Crouser ED Ruden E Julian MW Raman Sv. Resolution of abnormal cardiac MRI T2 signal following immune suppression for CS. J Invest Med. (2016) 64(6):1148–50. 10.1136/jim-2016-000144 27354042

[62] Tadamura E Yamamuro M Kubo S Kanao S Saga T Harada M Effectiveness of delayed enhanced MRI for identification of CS: comparison with radionuclide imaging. Am J Roentgenol. (2005) 185(1):110–5. 10.2214/ajr.185.1.01850110 15972409

[63] Bruder O Schneider S Pilz G van Rossum AC Schwitter J Nothnagel D 2015 update on acute adverse reactions to gadolinium based contrast agents in cardiovascular MR. Large multi-national and multi-ethnical population experience with 37788 patients from the EuroCMR registry. J Cardiovasc Magn Reson. (2015) 17(1):58. 10.1186/s12968-015-0168-3 26170152 PMC4501068

[64] Ayers-Ringler J McDonald JS Connors MA Fisher CR Han S Jakaitis DR Neurologic effects of gadolinium retention in the brain after gadolinium-based contrast agent administration. Radiology. (2022) 302(3):676–83. 10.1148/radiol.210559 34931861 PMC8893178

[65] Smedema JP Snoep G van Kroonenburgh MPG van Geuns RJ Dassen WRM Gorgels APM Evaluation of the accuracy of gadolinium-enhanced cardiovascular magnetic resonance in the diagnosis of CS. J Am Coll Cardiol. (2005) 45(10):1683–90. 10.1016/j.jacc.2005.01.047 15893188

[66] Shimada T Shimada K Sakane T Ochiai K Tsukihashi H Fukui M Diagnosis of CS and evaluation of the effects of steroid therapy by gadolinium-DTPA–enhanced magnetic resonance imaging. Am J Med. (2001) 110(7):520–7. 10.1016/S0002-9343(01)00677-5 11343665

[67] Yamada S Kubota K Kubota R Ido T Tamahashi N. High accumulation of fluorine-18-fluorodeoxyglucose in turpentine-induced inflammatory tissue. J Nucl Med. (1995) 36(7):1301–6. . 7790960

[68] Mochizuki T Tsukamoto E Kuge Y Kanegae K Zhao S Hikosaka K FDG Uptake and glucose transporter subtype expressions in experimental tumor and inflammation models. J Nucl Med. (2001) 42(10):1551–5. . 11585872

[69] Ishimaru S Tsujino I Takei T Tsukamoto E Sakaue S Kamigaki M Focal uptake on 18F-fluoro-2-deoxyglucose positron emission tomography images indicates cardiac involvement of sarcoidosis†. Eur Heart J. (2005) 26(15):1538–43. 10.1093/eurheartj/ehi180 15809286

[70] Bartlett ML Bacharach SL Voipio-Pulkki LM Dilsizian V. Artifactual inhomogeneities in myocardial PET and SPECT scans in normal subjects. J Nucl Med. (1995) 36(2):188–95. . 7830111

[71] Tadamura E Yamamuro M Kubo S Kanao S Hosokawa R Kimura T Multimodality imaging of CS before and after steroid therapy. Circulation. (2006) 113(20. 10.1161/CIRCULATIONAHA.105.594200 16717157

[72] Takeda N Yokoyama I Hiroi Y Sakata M Harada T Nakamura F Positron emission tomography predicted recovery of complete A-V nodal dysfunction in a patient with CS. Circulation. (2002) 105(9):1144–5. 10.1161/hc0902.103432 11877369

[73] Ambrosini V Zompatori M Fasano L Nanni C Nava S Rubello D 18F-FDG PET/CT for the assessment of disease extension and activity in patients with sarcoidosis. Clin Nucl Med. (2013) 38(4):e171–7. 10.1097/RLU.0b013e31827a27df 23429384

[74] Harisankar CNB Mittal BR Agrawal KL Abrar ML Bhattacharya A. Utility of high fat and low carbohydrate diet in suppressing myocardial FDG uptake. J Nucl Cardiol. (2011) 18(5):926–36. 10.1007/s12350-011-9422-8 21732228

[75] Williams G Kolodny GM. Suppression of myocardial ^18^F-FDG uptake by preparing patients with a high-fat, low-carbohydrate diet. Am J Roentgenol. (2008) 190(2):W151–6. 10.2214/AJR.07.2409 18212199

[76] Scholtens AM Verberne HJ Budde RPJ Lam MGEH. Additional heparin preadministration improves cardiac glucose metabolism suppression over low-carbohydrate diet alone in ^18^F-FDG PET imaging. J Nucl Med. (2016) 57(4):568–73. 10.2967/jnumed.115.166884 26659348

[77] Alavi A Gupta N Alberini JL Hickeson M Adam LE Bhargava P Positron emission tomography imaging in non-malignant thoracic disorders. Semin Nucl Med. (2002) 32(4):293–321. 10.1053/snuc.2002.127291 12524653

[78] Yodogawa K Seino Y Ohara T Takayama H Katoh T Mizuno K. Effect of corticosteroid therapy on ventricular arrhythmias in patients with CS. Ann Noninvasive Electrocardiol. (2011) 16(2):140–7. 10.1111/j.1542-474X.2011.00418.x 21496164 PMC6932647

[79] Tan JL Tan BEX Cheung JW Ortman M Lee JZ. Update on CS. Trends Cardiovasc Med. (2022). 10.1016/j.tcm.2022.04.007 35504422

[80] Kopřiva P Gřiva M Tüdös Z. Management of CS—a practical guide. Cor Vasa. (2018) 60(2):e155–64. 10.1016/j.crvasa.2017.05.012

[81] Milburn HJ Poulter LW Dilmec A Cochrane GM Kemeny DM. Corticosteroids restore the balance between locally produced Th1 and Th2 cytokines and immunoglobulin isotypes to normal in sarcoid lung. Clin Exp Immunol. (2003) 108(1):105–13. 10.1046/j.1365-2249.1997.d01-979.x PMC1904629 9097918

[82] Oswald-Richter KA Richmond BW Braun NA Isom J Abraham S Taylor TR Reversal of global CD4^+^ subset dysfunction is associated with spontaneous clinical resolution of pulmonary sarcoidosis. The Journal of Immunology. (2013) 190(11):5446–53. 10.4049/jimmunol.1202891 23630356 PMC3660530

[83] Isobe M Tezuka D. Isolated CS: clinical characteristics, diagnosis and treatment. Int J Cardiol. (2015) 182:132–40. 10.1016/j.ijcard.2014.12.056 25577749

[84] Timmers M Claeys MJ Vanhauwaert B Rivero-Ayerza M de Hondt G. CS: a diagnostic and therapeutic challenge. Acta Cardiol. (2018) 73(1):1–6. 10.1080/00015385.2017.1325633 28675086

[85] Kato Y Morimoto Si Uemura A Hiramitsu S Ito T Hishida H. Efficacy of corticosteroids in sarcoidosis presenting with atrioventricular block. Sarcoidosis Vasc Diffuse Lung Dis. (2003) 20(2):133–7. . 12870723

[86] Kolluri N Elwazir MY Rosenbaum AN Maklady FA AbouEzzeddine OF Kapa S Effect of corticosteroid therapy in patients with CS on frequency of venous thromboembolism. Am J Cardiol. (2021) 149:112–8. 10.1016/j.amjcard.2021.03.017 33757783

[87] Cronstein BN Aune TM. Methotrexate and its mechanisms of action in inflammatory arthritis. Nat Rev Rheumatol. (2020) 16(3):145–54. 10.1038/s41584-020-0373-9 32066940

[88] Nagai S Yokomatsu T Tanizawa K Ikezoe K Handa T Ito Y Treatment with methotrexate and low-dose corticosteroids in sarcoidosis patients with cardiac lesions. Intern Med. (2014) 53(5):427–33. 10.2169/internalmedicine.53.0794 24583430

[89] Cremers JP Drent M Bast A Shigemitsu H Baughman RP Valeyre D Multinational evidence-based world association of sarcoidosis and other granulomatous disorders recommendations for the use of methotrexate in sarcoidosis. Curr Opin Pulm Med. (2013) 19(5):545–61. 10.1097/MCP.0b013e3283642a7a 23880702

[90] Crouser ED Maier LA Wilson KC Bonham CA Morgenthau AS Patterson KC Diagnosis and detection of sarcoidosis. An official American thoracic society clinical practice guideline. Am J Respir Crit Care Med. (2020) 201(8):e26–51. 10.1164/rccm.202002-0251ST 32293205 PMC7159433

[91] Gilotra N Okada D Sharma A Chrispin J. Management of CS in 2020. Arrhythm Electrophysiol Rev. (2020) 9(4):182–8. 10.15420/aer.2020.09 33437485 PMC7788397

[92] Echt DS Liebson PR Mitchell LB Peters RW Obias-Manno D Barker AH Mortality and morbidity in patients receiving encainide, flecainide, or placebo. N Engl J Med. (1991) 324(12):781–8. 10.1056/NEJM199103213241201 1900101

[93] Jamilloux Y Cohen-Aubart F Chapelon-Abric C Maucort-Boulch D Marquet A Pérard L Efficacy and safety of tumor necrosis factor antagonists in refractory sarcoidosis: a multi-center study of 132 patients. Semin Arthritis Rheum. (2017) 47(2):288–94. 10.1016/j.semarthrit.2017.03.005 28392046

[94] Giblin GT Murphy L Stewart GC Desai AS di Carli MF Blankstein R CS: when and how to treat inflammation. Card Fail Rev. (2021) 7. 10.15420/cfr.2021.16 34950507 PMC8674699

[95] Kron J Sauer W Schuller J Bogun F Crawford T Sarsam S Efficacy and safety of implantable cardiac defibrillators for treatment of ventricular arrhythmias in patients with CS. EP Europace. (2013) 15(3):347–54. 10.1093/europace/eus316 23002195

[96] Yazaki Y Isobe M Hiroe M Morimoto Si Hiramitsu S Nakano T Prognostic determinants of long-term survival in Japanese patients with CS treated with prednisone. Am J Cardiol. (2001) 88(9):1006–10. 10.1016/S0002-9149(01)01978-6 11703997

[97] Jefic D Joel B Good E Morady F Rosman H Knight B Role of radiofrequency catheter ablation of ventricular tachycardia in CS: report from a multi-center registry. Heart Rhythm. (2009) 6(2):189–95. 10.1016/j.hrthm.2008.10.039 19187909

[98] Zeppenfeld K Tfelt-Hansen J de Riva M Winkel BG Behr ER Blom NA 2022 ESC guidelines for the management of patients with ventricular arrhythmias and the prevention of sudden cardiac death. Eur Heart J. (2022) 43(40):3997–4126. 10.1093/eurheartj/ehac262 36017572

[99] Bobbio E Björkenstam M Nwaru BI Giallauria F Hessman E Bergh N Short- and long-term outcomes after heart transplantation in CS and giant-cell myocarditis: a systematic review and meta-analysis. Clin Res Cardiol. (2022) 111(2):125–40. 10.1007/s00392-021-01920-0 34402927 PMC8816313

[100] Yager JEE Hernandez AF Steenbergen C Persing B Russell SD Milano C Recurrence of CS in a heart transplant recipient. J Heart Lung Transplant. (2005) 24(11):1988–90. 10.1016/j.healun.2005.02.016 16297811

